# Co‐amplified with PDGFRA, IGFBP7 is a prognostic biomarker correlated with the immune infiltrations of glioma

**DOI:** 10.1002/cam4.5187

**Published:** 2022-08-31

**Authors:** Haiwei Wang, Xinrui Wang, Liangpu Xu, Ji Zhang

**Affiliations:** ^1^ Fujian Maternity and Child Health Hospital, Fujian Medical University Fuzhou China; ^2^ Rui‐Jin Hospital, Shanghai Jiao Tong University School of Medicine Shanghai China

**Keywords:** glioblastoma, IGFBP7, immune infiltrations, lower grade glioma, PDGFRA

## Abstract

**Background:**

A subgroup of glioma carry genetic 4q12 amplification including platelet derived growth factor receptor α (PDGFRA) and insulin like growth factor binding protein 7 (IGFBP7). However, the prognosis of PDGFRA and IGFBP7 in glioma is unclear.

**Methods:**

The prognosis of PDGFRA and IGFBP7 was determined using cox regression and Kaplan–Meier survival analysis. Pathways associated with IGFBP7 were analyzed through gene set enrichment analysis (GSEA). Immune profiling of glioma was determined using “ESTIMATE” and “TIMER” database.

**Results:**

PDGFRA amplification or expression was not correlated with the outcomes of glioblastoma (GBM). IGFBP7 but not PDGFRA was over‐expressed in GBM. IGFBP7 over‐expression was correlated with the unfavorable outcomes of GBM. In lower grade glioma (LGG), PDGFRA over‐expression was not correlated with the unfavorable prognosis of LGG, while, IGFBP7 was a prognostic biomarker of LGG. LGG patients with IGFBP7 lower expressions had prolonged clinical overall survival. Combination of IDH mutation, LGG grade and IGFBP7 achieved even better prognostic effects in LGG. Moreover, IGFBP7 was over‐expressed in glioma patients with wild type IDH or with high grades. IGFBP7 over‐expression was correlated with the unfavorable outcomes of glioma. Furthermore, IGFBP7 was hypo‐methylated in GBM or LGG patients without IDH mutations. IGFBP7 hyper‐methylation was correlated with the lower overall survival of GBM or LGG. LGG patients with wild type IDH and with IGFBP7 hypo‐methylation demonstrated even worse prognosis. IGFBP7 was associated with multiple immune‐related signaling pathways in GBM or LGG. The stromal score, immune score and the infiltrations of immune cells were also correlated with IGFBP7 and the prognosis of LGG.

**Conclusions:**

IGFBP7 but not PDGFRA served an ideal prognostic marker and therapeutic target of glioma.

## Graphical abstract

PDGFRA and IGFBP7 were co‐amplifying in glioma. IGFBP7 but not PDGFRA served an ideal prognostic marker and therapeutic target of glioma. IGFBP7 was associated with the glioma immune infiltrations.

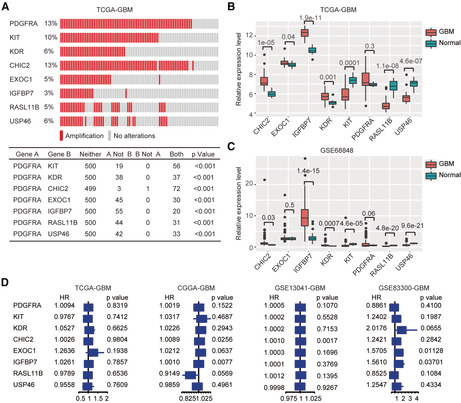

## 1 BACKGROUND

Glioma originated from the intracranial glial cells, represents the most aggressive subtype of brain tumors.^1^, ^2^ Despite the developments of surgery and adjuvant therapy, the clinical outcomes of glioma remain unsatisfied.^3^, ^4^ Glioma is heterogeneous and exhibits wide ranges of aggressiveness. Glioma is divided into grade 2, grade 3 glioma (lower grade glioma, LGG) and grade 4 glioma (glioblastoma, GBM).^5^ LGG and GBM share some common genetic abnormalities, like TP53 mutations and TERT promoter mutations.^6^, ^7^ However, amplification of epidermal growth factor receptor (EGFR) is detected in half of GBM patients,^6^, ^8^ while, LGG is characterized with mutations of IDH and 1p/19q co‐deletion.^9^ EGFR amplification is correlated with the unfavorable outcomes of GBM,^10^ while, IDH mutation represents a favorable prognostic marker of LGG.^7^, ^11^, ^12^ Identification of the molecular landscape of glioma is crucial to establish appropriate therapies and predict the clinical outcomes.

A subgroup of GBM patients also carries genetic amplifications at chromosomal 4q12 region which contains platelet derived growth factor receptor α (PDGFRA).^13^ As a tyrosine kinase receptor, PDGFRA interacts with PDGFs^14^ and controls the development of glial cells.^15^ PDGFRA is amplified or mutated in about 12% GBM patients.^16^, ^17^ PDGFRA amplification is correlated with the unfavorable outcomes of glioma.^18^, ^19^ PDGFRA expression is required for the cell proliferation of glioma.^20^ Moreover, PDGFRA is co‐expressed with EGFR and EGFR amplification requires PDGFRA signaling to promote the development of glioma.^21^ PDGFRA, IDH1 and EGFR alterations represent a distinct subtype of GBM.^22^ However, the functions of PDGFRA amplification and over‐expression in GBM or LGG should be further studied.

Except PDGFRA, vascular endothelial growth factor receptor 2 (VEGFR2, KDR) is also located at the chromosomal 4q12 region and co‐amplified with PDGFRA.^23^ KDR is a key mediator of endothelial proliferation, migration and angiogenesis.^24^ KDR over‐expression is correlated with the unfavorable outcomes of GBM and represents a promising therapeutic target in malignant GBM.^25^ More flanking genes at chromosomal 4q12 region are stem cell factor receptor (KIT), cysteine rich hydrophobic domain 2 (CHIC2), exocyst complex component 1 (EXOC1), insulin like growth factor binding protein 7 (IGFBP7), USP46 and RAS like family 11 member B (RASL11B).^13^ Like KDR, those PDGFRA co‐amplified genes are suspected driver genes to promote the progression of glioma. However, the expression and prognosis of KIT, CHIC2, EXOC1, IGFBP7, RASL11B or USP46 in glioma are unclear.

Using The Cancer Genome Atlas (TCGA) program,^26^ Chinese Glioma Genome Atlas (CGGA) database^27^, ^28^ and Gene Expression Omnibus (GEO) datasets, this study comprehensively analyzed the expression and prognosis of PDGFRA and its co‐amplified genes KIT, KDR, CHIC2, EXOC1, IGFBP7, RASL11B and USP46 in glioma. Among all those genes, IGFBP7 was particularly over‐expressed in glioma tissues. Moreover, expression and methylation levels of IGFBP7 were independent prognostic markers of GBM and LGG. Furthermore, IGFBP7 was correlated with the immune infiltrations of glioma. Our results suggested the potential prognostic biomarker and therapeutic target of IGFBP7 in GBM and LGG.

## 2 MATERIALS AND METHODS

### 2.1 Data sources

The TCGA‐GBM and TCGA‐LGG datasets were collected from UCSC Xena database.^26^ The CGGA datasets were collected from www.cgga.org.cn website.^27^, ^28^ GSE13041,^29^ GSE83300,^30^ GSE68848,^31^ GSE107850,^32^ GSE4412^33^ and GSE43378^34^ datasets was collected from NCBI‐GEO database. All the datasets were processed using R software.

### 2.2 Analysis of the genetic alterations using cbioportal

The profiling of genomic alterations of PDGFRA, IDH1 and IGFBP7 in TCGA datasets was analyzed using cbioportal. The prognostic effects of PDGFRA alterations were also analyzed using cbioportal. Cbioportal is an online platform for the analysis of cancer genomic alterations.^35^, ^36^

### 2.3 Univariate and multivariate cox regression

The prognosis of PDGFRA and its co‐amplified genes was analyzed by univariate or multivariate cox regression using R software “survival” package and “survminer” package. The forest plots were generated by “forestplot” or “ggforest” package. *p* value and hazard ratio (HR) were calculated using cox regression.

### 2.4 Kaplan–Meier survival analysis

The prognosis of PDGFRA and IGFBP7 was also analyzed by Kaplan–Meier survival analysis using R software “survival” package and “survminer” package. Patients with GBM or LGG were classified into “high” or “low” subgroup by the best cutoff points using “survminer” package. Log‐rank test was used to calculate the *p* value.

### 2.5 Volcano plots

Genes with *p* value <0.001 and fold change >2 thresholds were defined as the differentially expressed genes among IGFBP7 highly or lowly expressed glioma and were presented using volcano plots generated by “ggplot2” package in R software.

### 2.6 Functional annotation of IGFBP7 correlated genes

Functional annotations of genes correlated with IGFBP7 were carried out through The Database for Annotation, Visualization and Integrated Discovery (DAVID). DAVID is an enrichment web server for functional analysis of large gene list.^37^, ^38^ The pathways or transcription factors enriched in glioma patients with high IGFBP7 expressions were determined by Gene Set Enrichment Analysis (GSEA).^39^ Normalized enrichment score (NES) and *p* value were determined through 1000 of permutations.

### 2.7 Estimation of the stromal and immune score of glioma

The stromal and immune score of glioma were calculated using R software “ESTIMATE” package. “ESTIMATE” package determined the abundance of stromal cells and immune cells based on single sample GSEA.^40^ Spearman correlation was used to determine the correlations of the stromal or immune score with IGFBP7.

### 2.8 Immune infiltrations in glioma microenvironment

Analysis of the correlation of IGFBP7 with the immune infiltration in glioma microenvironment was carried out through “TIMER” (Tumor IMmune Estimation Resource) database.^41^, ^42^ The prognosis of immune cell infiltration was also determined by “TIMER” database.

### 2.9 Statistical analysis

Box plots were generated through GraphPad Prism or “ggplot2” package in R software. *p* value was calculated by two tails paired student's t test. *p* values <0.05 were considered statistically different.

## 3 RESULTS

### 3.1 PDGFRA amplification or expression is not significantly correlated with the prognosis of GBM

**FIGURE 1:**
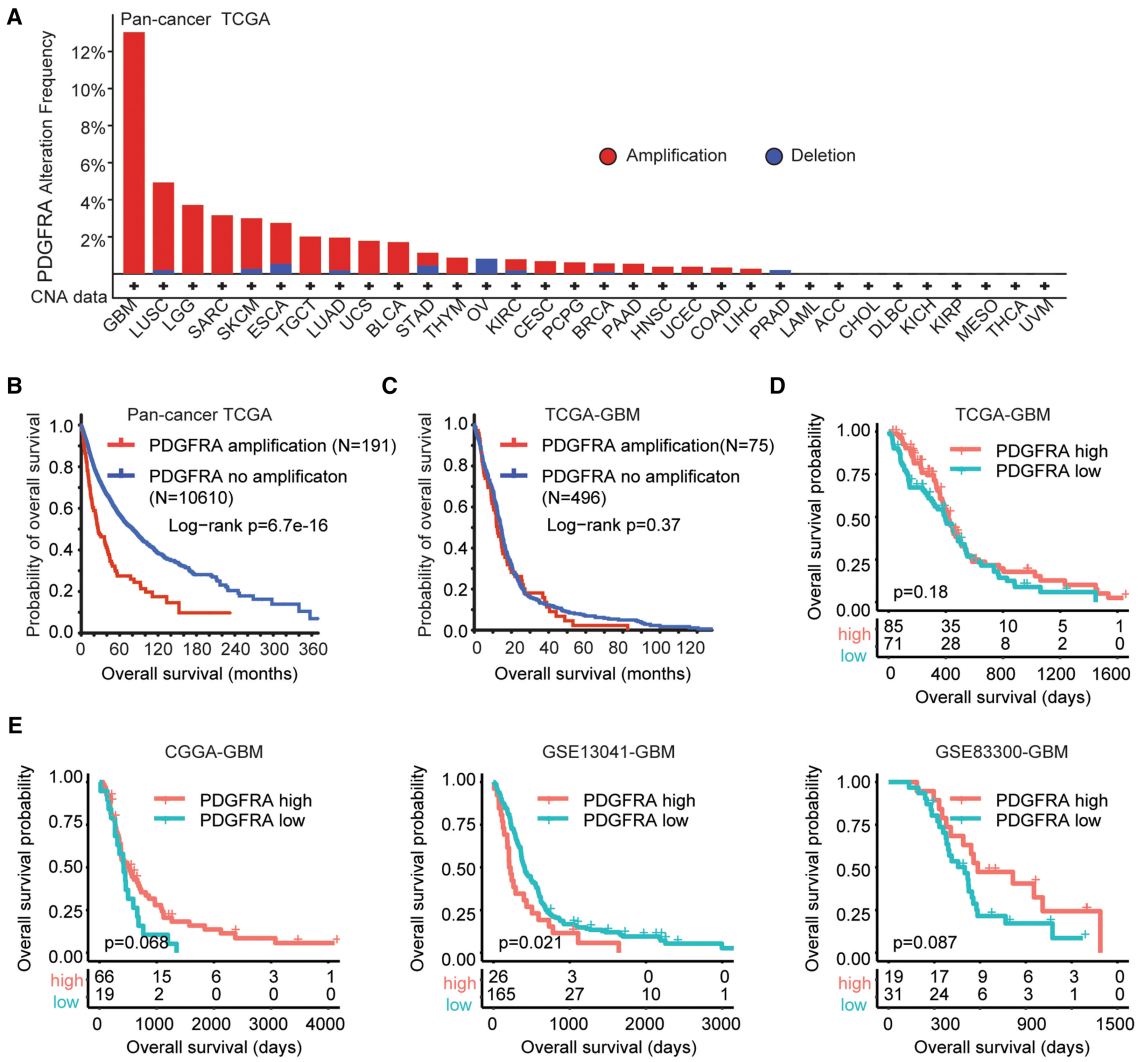
Prognostic relevance of PDGFRA amplification and expression. (A) Copy number alterations of PDGFRA in pan‐cancer TCGA datasets. (B) Overall survival of tumor patients without or with PDGFRA amplifications in pan‐cancer TCGA datasets. (C) Overall survival of GBM patients without or with PDGFRA amplifications in TCGA‐GBM dataset. (D) Overall survival of GBM patients with PDGFRA higher expressions or with PDGFRA lower expressions in TCGA‐GBM dataset. (E) Correlations of overall survival and PDGFRA expression were validated in CGGA‐GBM, GSE13041‐GBM and GSE83300‐GBM datasets

Based on the putative copy‐number alterations in TCGA pan‐cancer datasets, the amplification or deletion of PDGFRA was determined in 10,967 samples in 32 types of tumor. PDGFRA amplification was detected across broad types of tumor. PDGFRA was most frequently amplified in GBM. More than 10% GBM patients were with PDGFRA alterations (Figure 1A). PDGFRA amplification was also detected in lung squamous cell carcinoma (LUSC), LGG and sarcoma (SARC) in TCGA datasets (Figure 1A). Moreover, PDGFRA amplification was correlated with the shorted overall survival in pan‐cancer TCGA datasets (Figure 1B).

However, PDGFRA amplification was not correlated with the unfavorable outcomes of GBM. GBM patients without PDGFRA amplification had not significantly different clinical outcomes compared with GBM patients carrying PDGFRA amplification in TCGA‐GBM dataset (Figure 1C). Moreover, in TCGA‐GBM dataset, PDGFRA expression was neither significantly correlated with the prognosis of GBM (Figure 1D). The prognosis of PDGFRA was further tested in CGGA, GSE13041 and GSE83300 three independent GBM cohorts. In CGGA‐GBM and GSE83300‐GBM datasets, PDGFRA expression levels had no prognostic effects in GBM (Figure 1E). Only in GSE13041 dataset, GBM patients with lower PDGFRA expressions had prolonged overall survival than GBM patients with higher PDGFRA expressions (Figure 1E).

### 3.2 Expression and prognosis of PDGFRA co‐amplified genes in GBM

**FIGURE 2:**
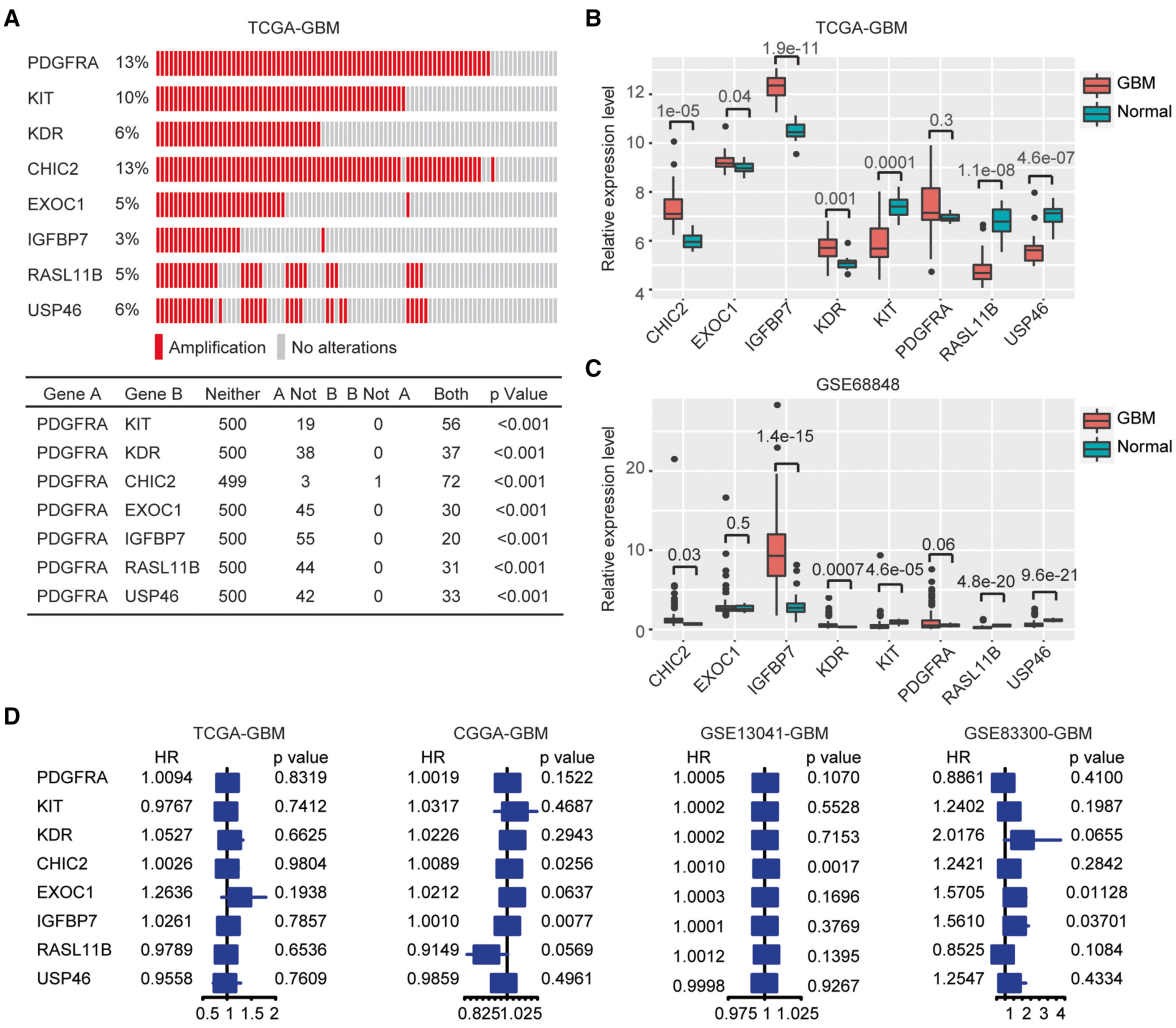
Expression and prognosis of PDGFRA co‐amplified genes. (A) Co‐occurrence of PDGFRA, KIT, KDR, CHIC2, EXOC1, IGFBP7, RASL11B, and USP46 amplification in TCGA‐GBM. (B) PDGFRA, KIT, KDR, CHIC2, EXOC1, IGFBP7, RASL11B and USP46 expressions in normal brain tissues and TCGA‐GBM tissues. (C) PDGFRA, KIT, KDR, CHIC2, EXOC1, IGFBP7, RASL11B and USP46 expression levels in normal brain tissues and GSE68848‐GBM tissues. (D) Associations of PDGFRA, KIT, KDR, CHIC2, EXOC1, IGFBP7, RASL11B and USP46 expression with GBM overall survival in TCGA‐GBM, CGGA‐GBM GSE13041‐GBM and GSE83300‐GBM datasets. *p* value and HR were determined through cox regression

PDGFRA is located at chromosomal 4q12 region. This region includes multiple genes which are co‐amplified with PDGFRA in GBM. In TCGA‐GBM dataset, KIT, KDR, CHIC2, EXOC1, IGFBP7, RASL11B and USP46 were significantly co‐amplified with PDGFRA (Figure 2A). GBM patients with KIT, KDR, CHIC2, EXOC1, IGFBP7, RASL11B or USP46 amplifications were also with PDGFRA amplifications (Figure 2A). Only one GBM patients with CHIC2 amplification was not with PDGFRA amplification (Figure 2A). Next, we determined the expression and prognosis of PDGFRA co‐amplified genes.

First, the expressions of PDGFRA, KIT, KDR, CHIC2, EXOC1, IGFBP7, RASL11B and USP46 were determined in GBM and normal brain tissues. Compared with normal brain tissues, CHIC2, IGFBP7 and KDR were over‐expressed in GBM in TCGA‐GBM dataset (Figure 2B). Particularly, IGFBP7 was most significantly up‐regulated in GBM (Figure 2B). However, in GBM, KIT, RASL11B and USP46 were down‐regulated (Figure 2B), and there were no significantly different PDGFRA expression between GBM and normal brain tissues in TCGA‐GBM dataset (Figure 2B). Moreover, compared with PDGFRA, KIT, KDR, CHIC2, EXOC1, RASL11B or USP46, the expression of IGFBP7 was highest in GBM in TCGA‐GBM dataset (Figure 2B).

The expressions of PDGFRA, KIT, KDR, CHIC2, EXOC1, IGFBP7, RASL11B and USP46 in GBM and normal brain tissues were confirmed in GSE68848 dataset. Consistent with TCGA‐GBM dataset, IGFBP7 was most significantly over‐expressed in GBM, contrast with other PDGFRA co‐amplified genes (Figure 2C). Also, compared with PDGFRA, KIT, KDR, CHIC2, EXOC1, RASL11B or USP46, the expression of IGFBP7 was highest in GBM in GSE68848 dataset (Figure 2C).

Next, we analyzed the prognosis of KIT, KDR, CHIC2, EXOC1, IGFBP7, RASL11B and USP46 in TCGA‐GBM dataset. Using univariate cox regression analysis, we found that KIT, KDR, CHIC2, EXOC1, IGFBP7, RASL11B or USP46 was not correlated with the clinical overall survival of GBM in TCGA‐GBM dataset (Figure 2D). The prognosis of KIT, KDR, CHIC2, EXOC1, IGFBP7, RASL11B or USP46 was further tested in CGGA, GSE13041 and GSE83300 three independent GBM cohorts. IGFBP7 was correlated with the clinical overall survival of GBM in CGGA‐GBM and GSE83300‐GBM datasets (Figure 2D). CHIC2 was correlated with the overall survival of GBM in CGGA‐GBM and GSE13041‐GBM datasets (Figure 2D). EXOC1 was correlated with the clinical overall survival of GBM in GSE83300‐GBM dataset (Figure 2D). However, other PDGFRA co‐amplified genes were not correlated with the clinical overall survival of GBM in CGGA‐GBM, GSE13041‐GBM or GSE83300‐GBM dataset (Figure 2D). Those results suggested that among the PDGFRA co‐amplified genes, IGFBP7 was a potential prognostic biomarker of GBM.

### 3.3 IGFBP7 over‐expression is correlated with the progression of GBM

**FIGURE 3:**
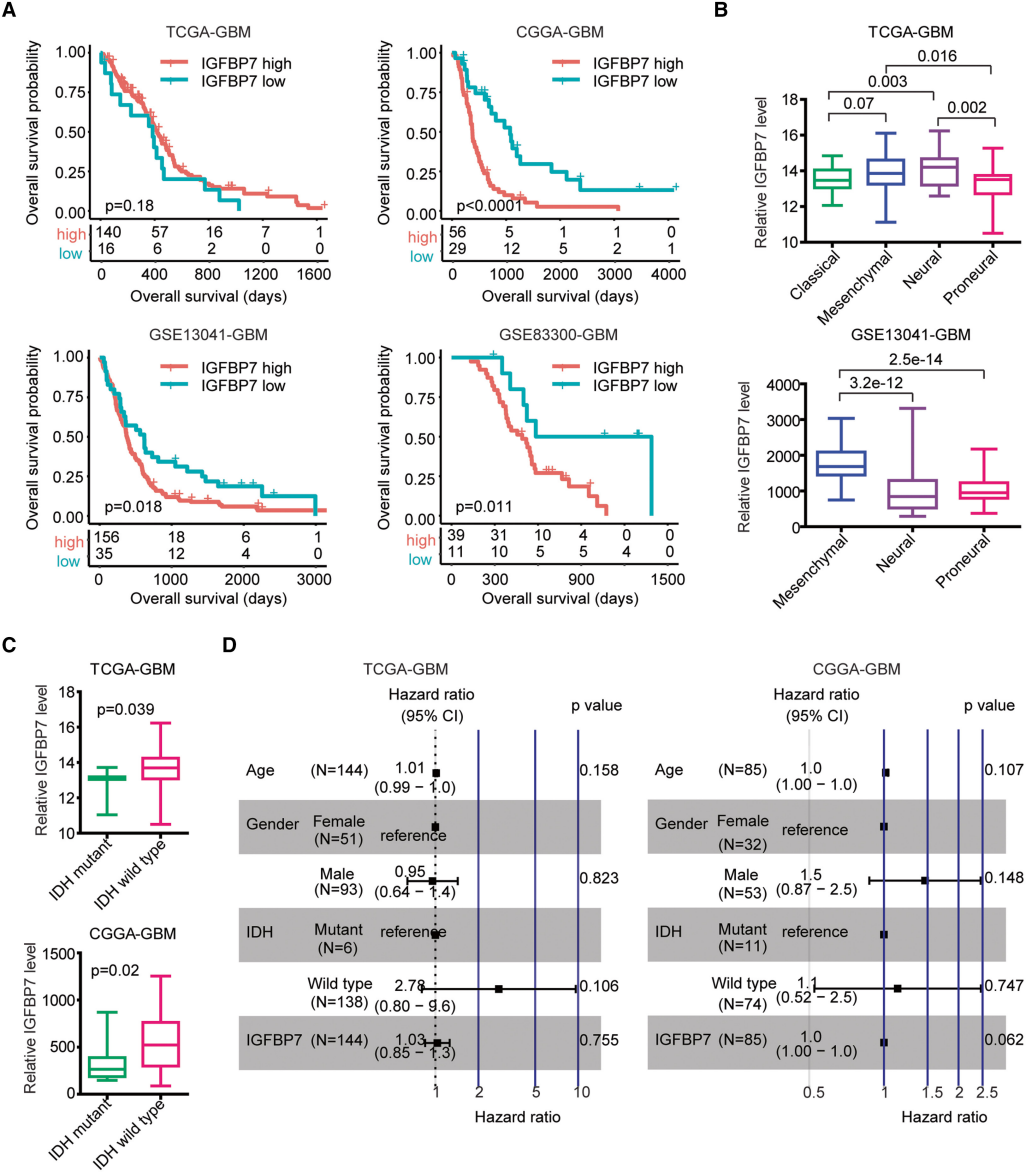
IGFBP7 over‐expression is correlated with the progression of GBM. (A) Correlations of IGFBP7 with GBM overall survival in TCGA‐GBM, CGGA‐GBM, GSE13041‐GBM and GSE83300‐GBM datasets were demonstrated through The Kaplan–Meier plots. (B) IGFBP7 expressions in GBM mesenchymal, classical, proneural and neural subtypes of TCGA‐GBM and GSE13041‐GBM. (C) IGFBP7 expressions in IDH mutant GBM patients or IDH non‐mutant GBM patients in TCGA‐GBM and CGGA‐GBM datasets. (D) Correlations of age of diagnosis, gender, IDH mutation and IGFBP7 expression in the predication of the GBM overall survival in TCGA‐GBM and CGGA‐GBM datasets. *p* value and HR were determined by multivariate cox regression

Furthermore, the prognosis of IGFBP7 in GBM was analyzed using Kaplan–Meier survival analysis. Although the prognosis of IGFBP7 in TCGA‐GBM dataset was not significant, GBM patients with IGFBP7 higher expression levels had shored overall survival compared with GBM patients with IGFBP7 lower expression levels in CGGA, GSE13041 and GSE83300 three independent GBM cohorts (Figure 3A).

GBM was classified into neural, mesenchymal, classical and proneural subtypes.^8^ Compared with the classical or proneural subtypes, IGFBP7 expression levels were higher in mesenchymal subtype of GBM in TCGA‐GBM dataset (Figure 3B). The higher IGFBP7 expression in mesenchymal subtype of GBM was validated in GSE13041‐GBM dataset (Figure 3B). All those results suggested the importance of IGFBP7 as an unfavorable marker in the prediction of the clinical outcome of GBM.

A subgroup of GBM patients also carries genetic mutations of IDH. IDH mutation confers the favorable outcomes of GBM. Compared with GBM patients with IDH mutations, IGFBP7 was over‐expressed in IDH wild type GBM patients in TCGA‐GBM and CGGA‐GBM datasets (Figure 3C). However, age, gender, IGFBP7 expression or IDH mutation was not independent prognostic marker of GBM, as determined by multivariate cox regression survival analysis (Figure 3D).

### 3.4 IGFBP7 over‐expression is correlated with the progression of LGG

**FIGURE 4:**
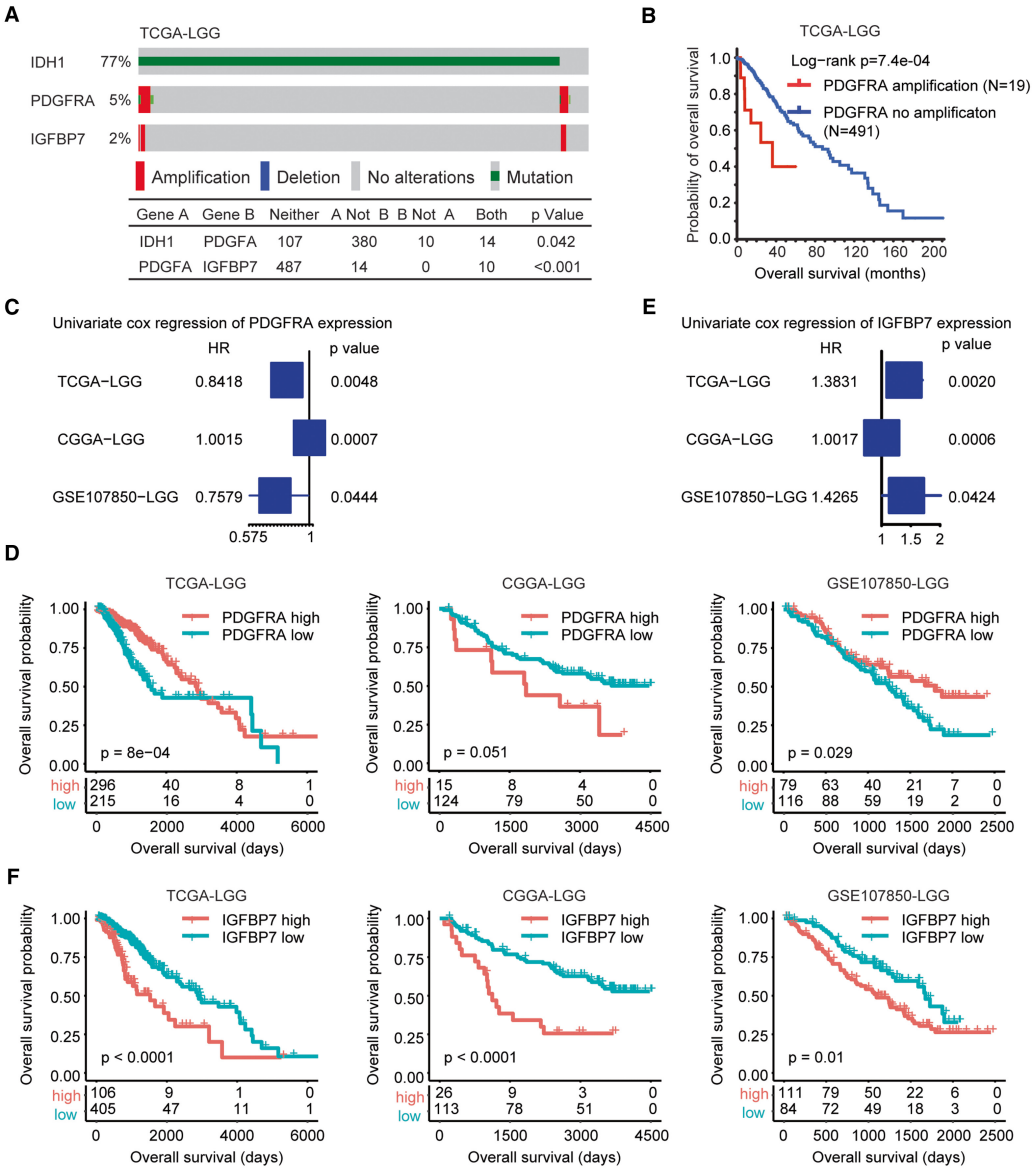
IGFBP7 over‐expression is correlated with the progression of LGG. (A) Oncoprint of IDH1 mutation, PDGFRA amplification and IGFBP7 amplification in TCGA‐LGG dataset. (B) Overall survival of LGG patients without or with PDGFRA amplifications in TCGA‐LGG dataset. (C) Forest plots revealed the associations of PDGFRA expression with LGG overall survival in TCGA‐LGG, CGGA‐LGG and GSE107850‐LGG datasets. (D) Overall survival of LGG patients with PDGFRA higher expressions or with PDGFRA lower expressions in TCGA‐LGG, CGGA‐LGG and GSE107850‐LGG datasets. (E) Forest plots revealed the associations of IGFBP7 expression with LGG overall survival in TCGA‐LGG, CGGA‐LGG and GSE107850‐LGG datasets. (F) Overall survival of LGG patients with IGFBP7 higher expressions or with IGFBP7 lower expressions in TCGA‐LGG, CGGA‐LGG and GSE107850‐LGG datasets

Compared with GBM, the frequency of PDGFRA amplification was lower in LGG (Figure 1A). LGG is characterized with IDH mutation. In TCGA‐LGG dataset, 77% LGG patients had IDH1 mutations, while, 5% LGG patients carried PDGFRA amplifications (Figure 4A). The IDH1 mutation and PDGFRA amplification co‐occurrence was low in TCGA‐LGG dataset (Figure 4A). However, like GBM, the co‐occurrence of PDGFRA and IGFBP7 amplification was statistically significant in LGG (Figure 4A). Ten LGG patients were with both PDGFRA and IGFBP7 alterations. Unlike GBM, LGG patients carrying PDGFRA amplifications had worse prognosis, contrast with PDGFRA non‐amplified LGG patients in TCGA‐LGG dataset (Figure 4B). However, the prognosis of PDGFRA expression was controversial. In TCGA‐LGG and GSE107850‐LGG datasets, higher expression of PDGFRA was a favorable prognostic factor, while, in CGGA‐LGG dataset, PDGFRA higher expression had unfavorable prognostic effects (Figure 4C). Indeed, LGG patients with PDGFRA higher expressions had prolonged overall survival than LGG patients with PDGFRA lower expressions in TCGA‐LGG and GSE107850‐LGG datasets (Figure 4D).

On the contrary, IGFBP7 was a more significant prognostic factor of LGG. IGFBP7 expression was significantly correlated with the prognosis of LGG in TCGA, CCGA and GSE107850 three independent LGG cohorts (Figure 4E). Moreover, the Kaplan–Meier survival analysis validated the unfavorable prognosis of IGFBP7 in LGG. LGG patients with IGFBP7 lower expression levels had prolonged overall survival contrast with IGFBP7 highly expressed LGG patients in TCGA‐LGG, CCGA‐LGG and GSE107850‐LGG datasets (Figure 4F).

### 3.5 Independent prognostic effects of IGFBP7 in LGG

**FIGURE 5:**
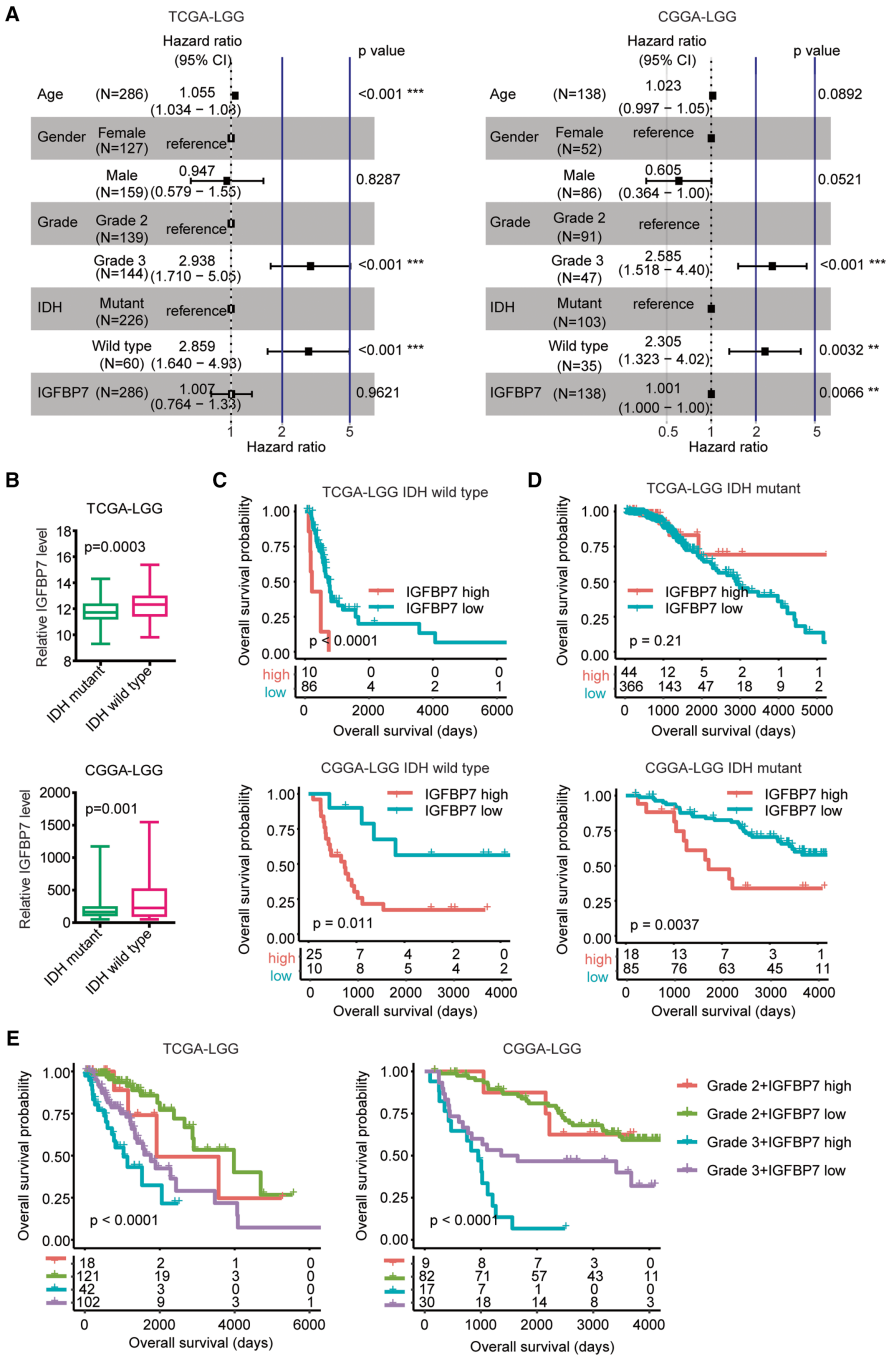
Independent prognostic effects of IGFBP7 in LGG. (A) Correlations of age of glioma diagnosis, gender, glioma grade, IDH mutation and IGFBP7 expression with LGG clinical overall survival in TCGA‐LGG and CGGA‐LGG datasets were determined by multivariate cox regression. (B) IGFBP7 expression levels in IDH mutant LGG patients or IDH wild type LGG patients in TCGA‐LGG and CGGA‐LGG datasets. (C) Associations of IGFBP7 with clinical overall survival of IDH wild type LGG patients in TCGA‐LGG and CGGA‐LGG datasets. (D) Associations of IGFBP7 with clinical overall survival of IDH mutant LGG patients in TCGA‐LGG and CGGA‐LGG datasets. (E) Overall survival of grade 2 IGFBP7 highly expressed LGG patients, grade 3 IGFBP7 highly expressed LGG patients, grade 2 IGFBP7 lowly expressed LGG patients and grade 3 IGFBP7 lowly expressed LGG patients in TCGA‐LGG and CGGA‐LGG datasets

Age of diagnosis, gender, IDH mutation and tumor grade were known prognostic markers of LGG.^43^ Next, we determined the relationships of IGFBP7 with age of diagnosis, gender, IDH mutation and tumor grade. Multivariate cox regression analysis demonstrated that LGG grade and IDH mutation were independent prognostic biomarker in TCGA‐LGG and CGGA‐LGG datasets (Figure 5A). Also, in TCGA‐LGG dataset, age was an independent prognostic biomarker, and in CGGA‐LGG dataset, IGFBP7 was an independent prognostic biomarker (Figure 5A).

IDH non‐mutant and IDH mutant LGG patients had significantly different clinical prognosis and gene expression profiling. IGFBP7 expression levels in LGG patients without or with IDH mutations were studied. IGFBP7 expression levels were higher in LGG patients without IDH mutations in TCGA‐LGG and CGGA‐LGG datasets (Figure 5B). Moreover, IGFBP7 had prognostic significance in LGG patients with non‐mutant IDH. LGG patients with wild type IDH and with higher IGFBP7 expressions had further unfavorable prognosis in TCGA‐LGG and CGGA‐LGG datasets (Figure 5C). IDH mutant LGG patients with lower IGFBP7 expressions had further favorable clinical outcomes in CGGA‐LGG dataset (Figure 5D), while, in IDH mutant LGG, the prognosis of IGFBP7 was not significant in TCGA‐LGG dataset (Figure 5D).

Furthermore, the synergistic prognostic effects of IGFBP7 and LGG grade were determined. LGG grade 3 patients with IGFBP7 higher expressions had the worst clinical overall survival in TCGA‐LGG and CGGA‐LGG datasets (Figure 5E), while, LGG grade 2 patients with IGFBP7 lower expressions had the best clinical prognosis (Figure 5E). Those results highlighted the combination of IGFBP7 with IDH mutation, grade in the prognosis of LGG.

### 3.6 Expression and prognosis of IGFBP7 in glioma

**FIGURE 6:**
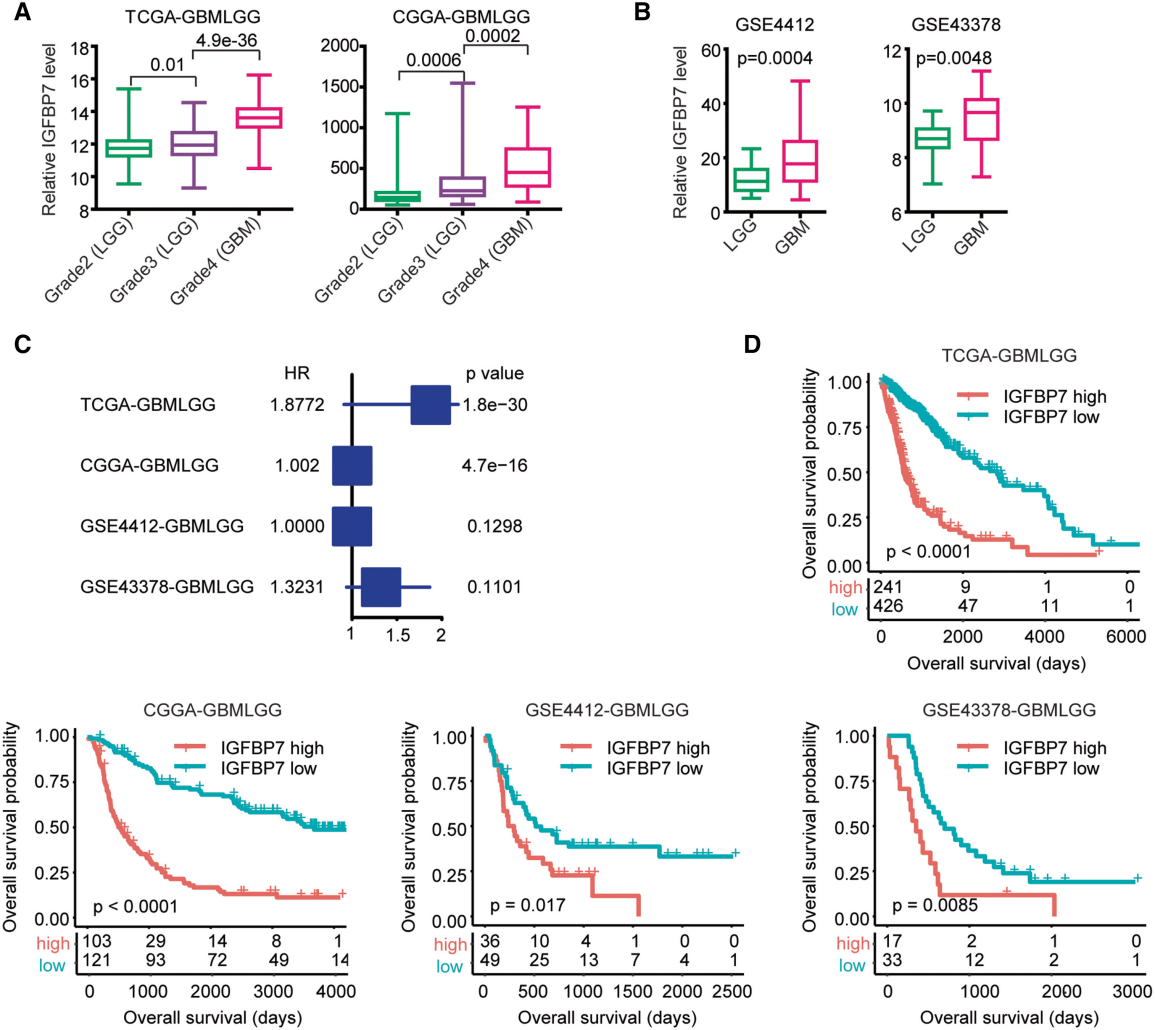
Expression and prognosis of IGFBP7 in glioma. (A) IGFBP7 expression levels in grade 2, 3 and 4 glioma patients. (B) IGFBP7 expression levels in GBM and LGG patients in GSE4412 and GSE43378 datasets. (C) Associations of IGFBP7 and overall survival of glioma in TCGA‐GBMLGG, CGGA‐GBMLGG, GSE4412‐GBMLGG and GSE43378‐GBMLGG. (D) Associations of IGFBP7 and overall survival of glioma in TCGA‐GBMLGG, CGGA‐GBMLGG, GSE4412‐GBMLGG and GSE43378‐GBMLGG datasets

The IGFBP7 expression levels in different grades of glioma were also tested. Contrasts with grade 2, IGFBP7 was up‐regulated in TCGA and CGGA grade 3 LGG patients (Figure 6A). IGFBP7 was further up‐regulated in TCGA and CGGA grade 4 GBM patients (Figure 6A). In GSE4412 and GSE43378 datasets, IGFBP7 was over‐expressed in GBM compared with LGG patients (Figure 6B).

Moreover, IGFBP7 was a prognostic biomarker of glioma and IGFBP7 was correlated with the overall survival of glioma in TCGA‐GBMLGG, CCGA‐GBMLGG, GSE4412‐GBMLGG and GSE43378‐GBMLGG four independent glioma cohorts (Figure 6C). Moreover, the Kaplan–Meier survival analysis validated the unfavorable prognostic effects of IGFBP7 in glioma. Glioma patients with IGFBP7 lower expressions had prolonged overall survival compared with glioma patients with IGFBP7 higher expressions in TCGA‐GBMLGG, CCGA‐GBMLGG, GSE4412 ‐GBMLGG and GSE43378 ‐GBMLGG datasets (Figure 6D).

### 3.7 IGFBP7 hypo‐methylation is correlated with the progression of glioma

**FIGURE 7:**
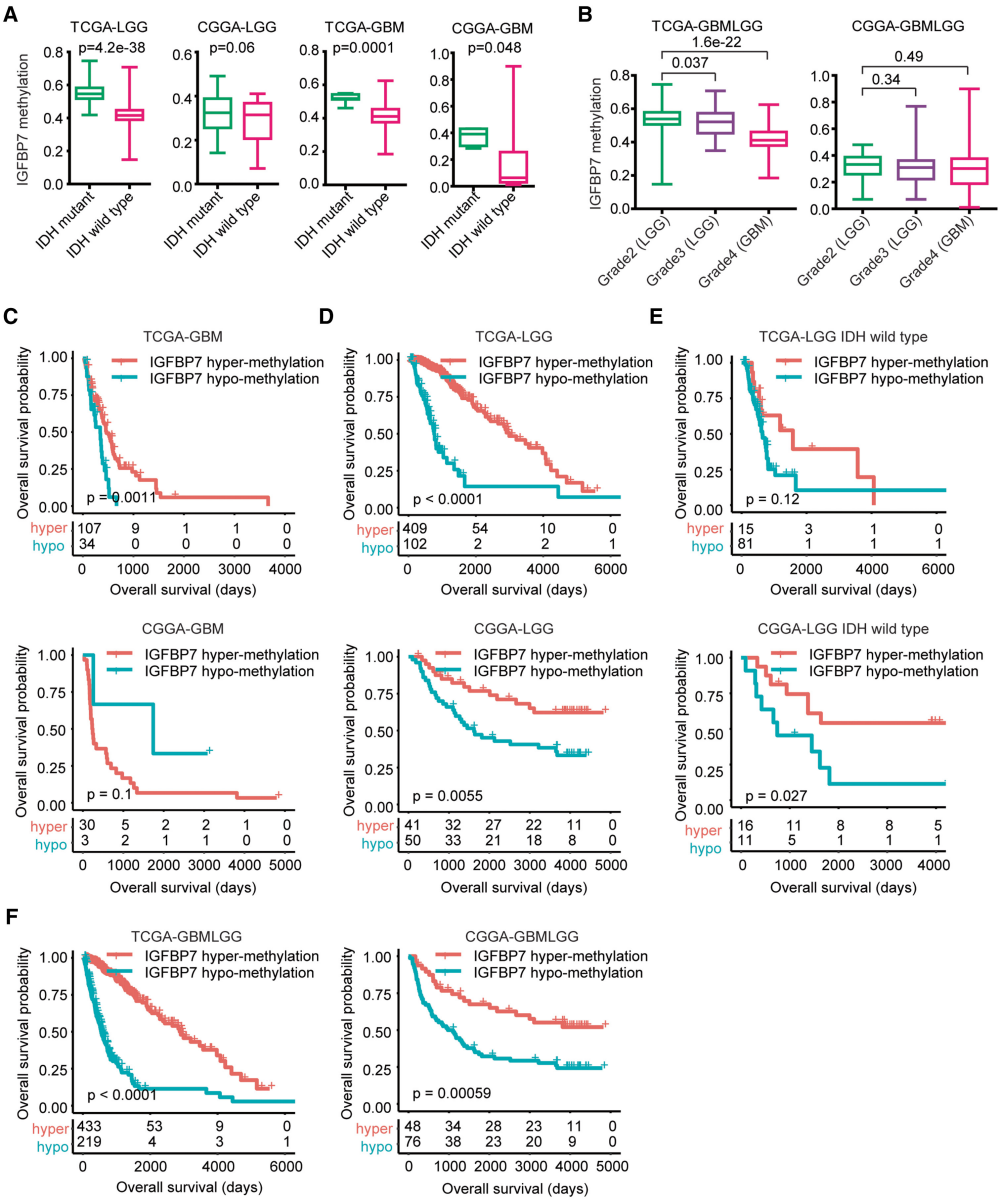
IGFBP7 hypo‐methylation is correlated with the progression of glioma. (A) IGFBP7 methylation levels in LGG or GBM patients without or with IDH mutations. (B) Methylation levels of IGFBP7 in grade 2, 3 or 4 glioma patients. (C) Clinical outcomes of GBM patients with IGFBP7 hyper‐methylation or with IGFBP7 hypo‐methylation in TCGA‐GBM and CGGA‐GBM datasets. (D) Overalls survival of IGFBP7 hyper‐methylated LGG patients and IGFBP7 hypo‐methylated LGG patients in TCGA‐LGG and CGGA‐LGG datasets. (E) Overalls survival of IGFBP7 hypo‐methylated IDH wild type LGG patients and IGFBP7 hyper‐methylated IDH wild type LGG patients in TCGA‐LGG and CGGA‐LGG datasets. (F) Overalls survival of IGFBP7 hypo‐methylated glioma patients and IGFBP7 hyper‐methylated glioma patients in TCGA‐GBMLGG and CGGA‐GBMLGG datasets

The alterations of IGFBP7 methylation in glioma were also contributing to the unfavorable prognostic significance of IGFBP7. First, IGFBP7 was hyper‐methylated in LGG patients with IDH mutations in TCGA‐LGG dataset (Figure 7A). Also, contrast with GBM patients without IDH mutations, IGFBP7 was hyper‐methylated in IDH mutated GBM patients in TCGA‐GBM and CGGA‐GBM datasets (Figure 7A). Furthermore, contrast with grade 2, IGFBP7 was hypo‐methylated in TCGA grade 3 LGG patients and further hypo‐methylated in TCGA grade 4 GBM patients (Figure 7B).

Next, the prognosis of IGFBP7 methylation was determined. IGFBP7 hyper‐methylated GBM patients had higher overall survival compared with IGFBP7 hypo‐methylated GBM patient in TCGA‐GBM, but not in CGGA‐GBM dataset (Figure 7C). And IGFBP7 hyper‐methylated LGG patients had prolonged overall survival than IGFBP7 hypo‐methylated LGG patients in TCGA‐LGG and CGGA‐LGG datasets (Figure 7D). Moreover, IGFBP7 methylation was correlated with the clinical outcomes of IDH wild type LGG patients in CGGA‐LGG, but not in TCGA‐LGG dataset (Figure 7E). At last, we showed the prognosis of IGFBP7 methylation in glioma. Similarly, IGFBP7 hyper‐methylated glioma had higher clinical overall survival compared with IGFBP7 hypo‐methylated glioma in TCGA‐GBMLGG and CGGA‐GBMLGG datasets (Figure 7F).

### 3.8 Identification of genes associated with IGFBP7

**FIGURE 8:**
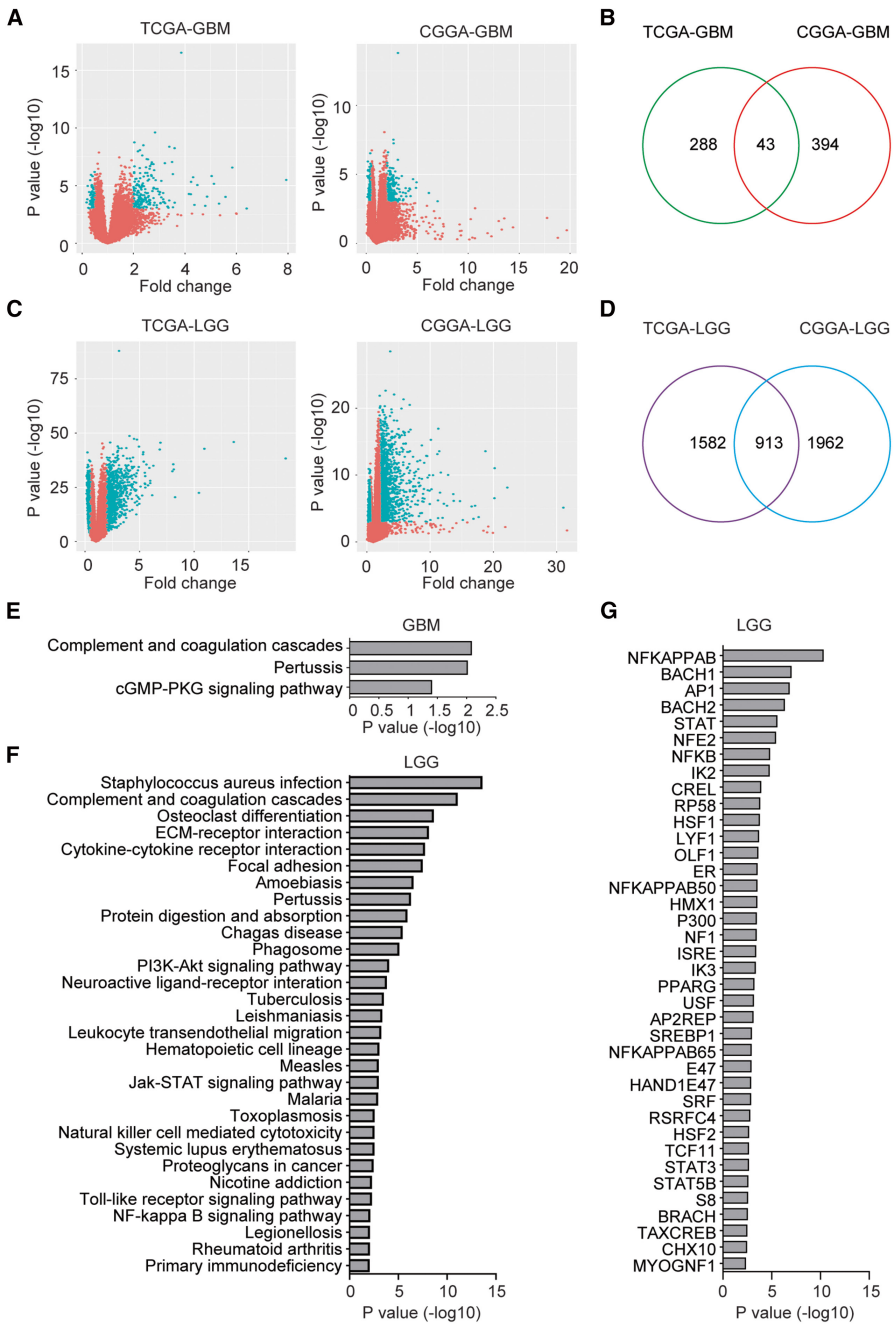
Identification of genes associated with IGFBP7. (A) Volcano plots showed the changed genes in IGFBP7 highly expressed GBM patients in TCGA‐GBM and CGGA‐GBM datasets. (B) Overlapped differentially expressed genes in IGFBP7 highly expressed GBM patients in TCGA‐GBM and CGGA‐GBM datasets. (C) Volcano plots showed the differentially expressed genes in IGFBP7 highly expressed LGG patients in TCGA‐LGG and CGGA‐LGG datasets. (D) Overlapped differentially expressed genes in IGFBP7 highly expressed LGG patients in TCGA‐LGG and CGGA‐LGG datasets. (E) Signaling pathways associated with IGFBP7 in GBM patients were determined by DAVID online tools. (F) Signaling pathways associated with IGFBP7 in LGG patients. (G) Transcription factors associated with IGFBP7 in LGG patients

To further demonstrate the functions of IGFBP7, we identified the differentially expressed genes between IGFBP7 highly expressed and lowly expressed glioma in TCGA and CGGA datasets. Using *p* value <0.001 and fold change >2 thresholds, 331 genes and 437 genes were changed in GBM patients with higher IGFBP7 expressions in TCGA‐GBM dataset and CGGA‐GBM dataset, respectively (Figure 8A). 43 genes were commonly associated with IGFBP7 in TCGA‐GBM and CGGA‐GBM datasets (Figure 8B). Similarly, 2495 genes and 2875 genes were changed in TCGA‐LGG and CGGA‐LGG datasets with higher IGFBP7 expressions, respectively (Figure 8C). 913 genes were commonly correlated with IGFBP7 in TCGA‐LGG and CGGA‐LGG datasets (Figure 8D).

Using DAVID online annotation, functional relevance of IGFBP7 correlated genes in glioma was determined. IGFBP7 was associated with several immune‐related signaling pathways in GBM and LGG. Genes differentially expressed in GBM patients with higher IGFBP7 expressions were associated with complement and coagulation cascades pathway (Figure 8E). Genes differentially expressed in LGG patients with higher IGFBP7 expressions were also significantly associated with complement and coagulation cascades pathway (Figure 8F). Moreover, cytokine‐cytokine receptor interaction, natural killer cell mediated cytotoxicity and NF‐κB signaling pathway were also associated with IGFBP7 in LGG patients (Figure 8F). Consistently, transcription factor NF‐κB was associated with IGFBP7 in LGG patients (Figure 8G).

### 3.9 Immune‐related pathways and NF‐κB transcription factor are correlated with IGFBP7

**FIGURE 9:**
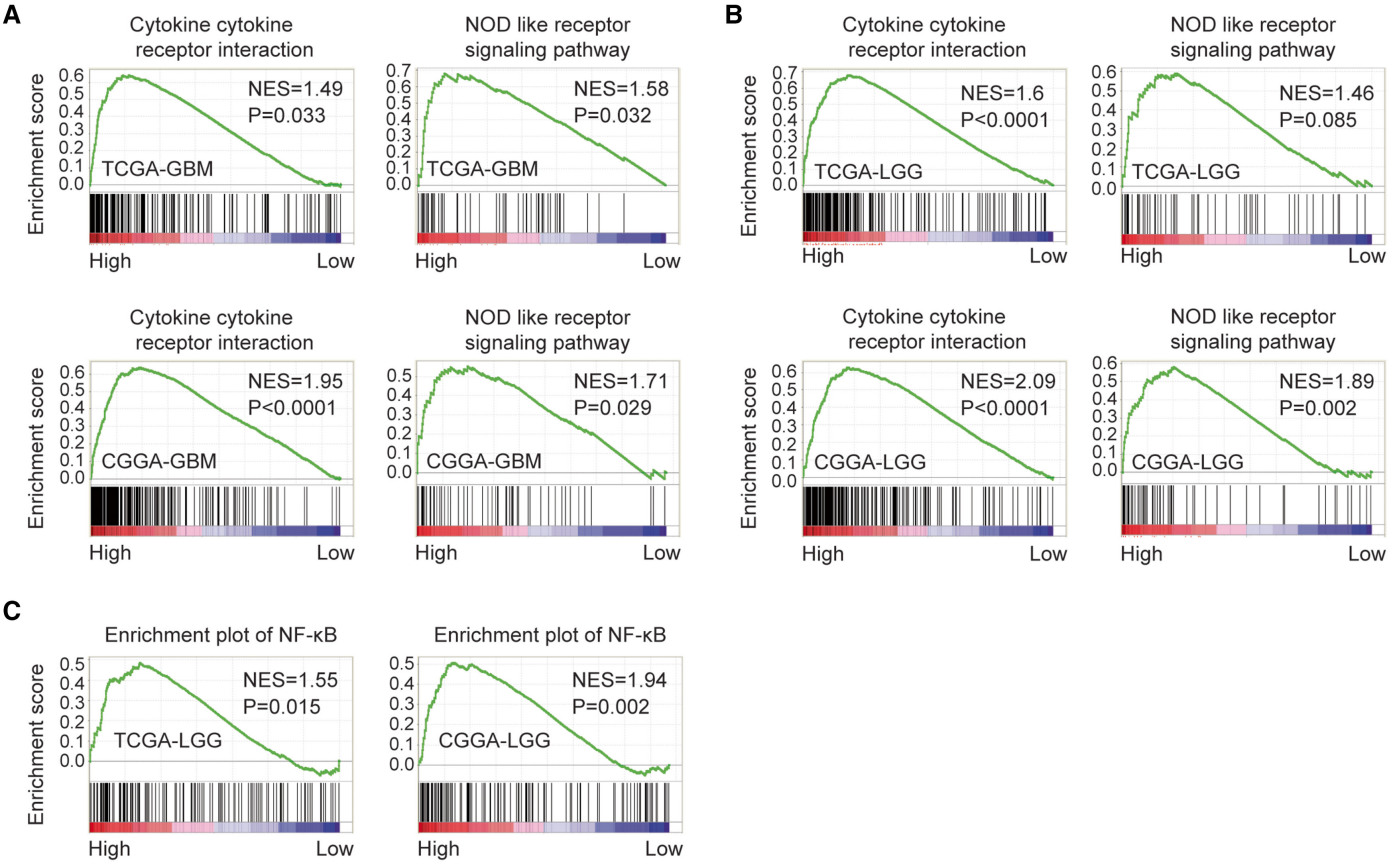
Immune‐related pathways and NF‐κB transcription factor are correlated with IGFBP7. (A) Enriched signaling pathways in GBM patients with higher IGFBP7 expressions or lower IGFBP7 expressions in TCGA‐GBM and CGGA‐GBM datasets. (B) Enriched signaling pathways in LGG patients with higher IGFBP7 expressions or lower IGFBP7 expressions in TCGA‐LGG and CGGA‐LGG datasets. (C) Enrichment of NF‐κB in LGG patients with higher IGFBP7 expressions or lower IGFBP7 expressions in TCGA and CGGA datasets.

The pathways associated with IGFBP7 in glioma were further determined through GSEA assay. In TCGA‐GBM and CCGA‐GBM datasets, GBM patients with higher IGFBP7 expressions were positively correlated with cytokine cytokine receptor interaction and NOD like receptor signaling pathway (Figure 9A). LGG patients with higher IGFBP7 expressions were also positively correlated with cytokine cytokine receptor interaction and NOD like receptor signaling pathway in TCGA‐LGG and CCGA‐LGG datasets (Figure 9B). Moreover, LGG patients with higher IGFBP7 expressions were also positively correlated with NF‐κB transcription factor in TCGA‐LGG and CCGA‐LGG datasets (Figure 9C). From DAVID and GSEA analysis, our results revealed that IGFBP7 expression was associated with the immune‐related pathways and NF‐κB transcription factor in glioma.

### 3.10 Correlations of IGFBP7 and immune infiltration of glioma

**FIGURE 10:**
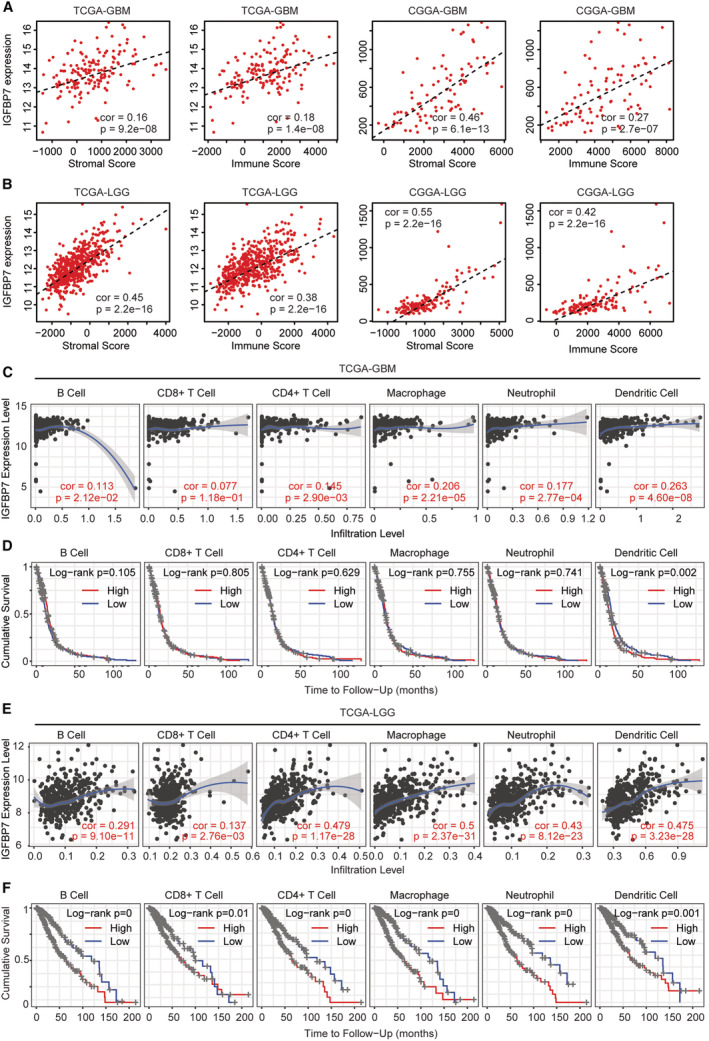
Correlations of IGFBP7 and immune infiltration of glioma. (A) Spearman correlations of IGFBP7 with stromal and immune score in TCGA‐GBM and CGGA‐GBM datasets. (B) Spearman correlations of IGFBP7 with stromal and immune score in TCGA‐LGG and CGGA‐LGG datasets. (C) Spearman correlations of IGFBP7 and the infiltrations of six immune‐related cell types in GBM patients in TCGA‐GBM dataset. (D) Prognosis of the infiltrations of six different immune cells in TCGA‐GBM dataset. (E) Spearman correlations of IGFBP7 and the infiltrations of six immune‐related cell types in TCGA‐LGG dataset. (F) Prognosis of the infiltrations of six different immune cells in TCGA‐LGG dataset

Immune signature was correlated with the outcomes and the immunotherapeutic responses of glioma.^44^ Next, we tested the correlations of IGFBP7 and immune infiltration of glioma. The stromal score, immune score and estimated score of GBM and LGG were calculated through “ESTIMATE” algorithm in TCGA and CGGA datasets. The stromal score as well as the immune score were associated with IGFBP7 expression levels in TCGA‐GBM and CGGA‐GBM datasets (Figure 10A). Also in LGG patients, the stromal score and the immune score were associated with IGFBP7 expression levels in TCGA‐LGG and CGGA‐LGG datasets (Figure 10B).

By studying the immune associated cell infiltrations using “TIMER” database in TCGA‐GBM dataset, we found that the dendritic cell infiltration was most correlated with IGFBP7 in GBM patients (Figure 10C). Moreover, higher dendritic cell infiltration was correlated with the unfavorable outcomes of GBM (Figure 10D). Infiltrations of other immune‐related cells, like B cell, CD8+ T cell, CD4+ T cell, macrophage cell and neutrophil cell were not correlated with IGFBP7 in TCGA‐GBM dataset (Figure 10C). Also, the infiltrations of B cell, CD8+ T cell, CD4+ T cell, macrophage cell or neutrophil cell were not correlated with the overall survival of GBM (Figure 10D).

In TCGA‐LGG dataset, the infiltrations of B cell, CD8+ T cell, CD4+ T cell, macrophage cell, neutrophil cell and dendritic cell were all statistically correlated with IGFBP7 (Figure 10E). Moreover, higher infiltrations of B cell, CD8+ T cell, CD4+ T cell, macrophage cell, neutrophil cell or dendritic cell were correlated with the unfavorable overall survival of LGG (Figure 10F). All those results highlighted the correlations of IGFBP7 and immune infiltration of glioma.

## 4 DISCUSSION

PDGFRA amplification is suggested as an unfavorable prognostic biomarker and a therapeutic target of GBM and LGG.^18^, ^19^ Multiple agents have been developed by targeting PDGFRA, including tyrosine kinase inhibitor dasatinib.^45^ However, our results provided some doubt about the prognostic and therapeutic roles of PDGFRA in glioma. In our results, the prognosis of PDGFRA amplification and expression was not significant in GBM. Also, over‐expression of PDGFRA was not correlated with the unfavorable outcomes of LGG. Moreover, there were no significantly different PDGFRA expressions between GBM and normal brain tissues in TCGA dataset. Our results were consistent with the clinical observations that, single inhibition of PDGFRA by dasatinib had failed to improve the clinical outcomes of glioma, even in glioma patients with PDGFRA amplifications or over‐expressions.^46^, ^47^ So, the detailed functions of PDGFRA amplification and over‐expression in GBM or LGG should be further illustrated.

Among the seven genes co‐amplified with PDGFRA at chromosomal 4q12 region, IGFBP7 was most significantly correlated with the progression of GBM and LGG. IGFBP7 is mainly produced by endothelial cells in glioma tissues^48^ and associated with the density of tumor vessels.^49^ IGFBP7 also mediates the angiogenesis of GBM by modulating Smad‐2‐dependent TGFβ signaling^50^ and regulates the growth of glioma cells.^51^ Contrast with the previous results that IGFBP7 lower expression was correlated with the unfavorable prognosis of glioma,^52^ our results demonstrated that IGFBP7 was over‐expressed in GBM and higher expression of IGFBP7 was correlated with the unfavorable prognosis of GBM. Moreover, LGG patients with lower IGFBP7 expressions had prolonged overall survival. IGFBP7 represented an independent prognostic biomarker. Combination of IGFBP7, IDH mutation and tumor grade achieved better prognostic effects of LGG. In glioma patients, higher expressions of IGFBP7 were also associated with the worse clinical outcomes. IGFBP7 was higher in glioma patients with wild type IDH or with higher grades. So, compared with PDGFRA, IGFBP7 was a more suitable prognostic and therapeutic target of glioma. And in PDGFRA driven glioma, inhibition of IGFBP7 may improve the efficacy of PDGFRA inhibitors.

Except gain of DNA copy number, altered DNA methylation of IGFBP7 was also contributing to the unfavorable prognostic effects of IGFBP7 in glioma. Compared with IDH mutant GBM or LGG patients, IGFBP7 was hypo‐methylated in IDH wild type GBM or LGG patients. IGFBP7 hyper‐methylated GBM patients had prolonged overall survival compared with IGFBP7 hypo‐methylated GBM patients. Moreover, LGG patients with IGFBP7 hyper‐methylation had more favorable prognosis compared with LGG patients with IGFBP7 hypo‐methylation. Furthermore, IGFBP7 hyper‐methylated IDH wild type LGG patients had shorted overall survival compared with IGFBP7 hypo‐methylated IDH wild type LGG patients. Overall, IGFBP7 amplification, IGFBP7 hypo‐methylation and IDH mutation were combined to contribute to the malignant roles of IGFBP7 in glioma.

IGFBP7 could influence the immune microenvironment of glioma.^53^, ^54^, ^55^ Previous results suggested that IGFBP7 was correlated with the immune cell infiltration of gastric cancer.^56^ Here, our results revealed that IGFBP7 was positively correlated with several immune‐related signaling pathways. The stromal and immune scores were also correlated with IGFBP7 in glioma patients, particular in LGG patients. Moreover, the infiltrations of B cell, CD8+ T cell, CD4+ T cell, macrophage cell, neutrophil cell and dendritic cell were all correlated with IGFBP7 and the prognosis of LGG. Those results suggested that IGFBP7 was an immune‐therapeutic target of glioma.

To our best knowledge, this was the first integrated and comprehensive study of the chromosomal 4q12 amplicon in glioma. Through analysis of the DNA amplification, gene expression, DNA methylation of IGFBP7, our results suggested that compared with other genes in 4q12 amplicon, IGFBP7 served as ideal prognostic marker of glioma. However, our analysis was used online datasets and the conclusions were short of experimental validations. Next, large glioma cohorts should be further studied to confirm the prognostic roles of IGFBP7. Also the relationships between IGFBP7 and immune microenvironment of glioma were needed further studies.

## 5 CONCLUSIONS

PDGFRA and IGFBP7 were co‐amplifying in GBM and LGG. IGFBP7 but not PDGFRA served an ideal prognostic marker and therapeutic target of glioma. IGFBP7 was up‐regulated in GBM. IGFBP7 over‐expression was correlated with the worse outcomes of GBM or LGG. IGFBP7 hyper‐methylation was correlated with the prolonged overall survival of GBM or LGG. IGFBP7 was associated with the glioma immune infiltrations.

## AUTHOR CONTRIBUTIONS

HW designed the working flow, analyzed the data and wrote the paper. XW and LX collected and analyzed the data. JZ supervised the study.

## FUNDING INFORMATION

The present study was supported by Natural Science Foundation of Fujian province (grant nos.2020 J01337).

## CONFLICT OF INTEREST

The authors declare that they have no conflict of interest.

## ETHICS APPROVAL AND CONSENT TO PARTICIPATE

Not applicable.

## CONSENT FOR PUBLICATION

Not applicable.

## Data Availability

The analyzed datasets during current study are available in TCGA, CGGA and GEO databases.
